# Exploring interprofessional collaboration during the integration of diabetes teams into primary care

**DOI:** 10.1186/s12875-016-0407-1

**Published:** 2016-02-01

**Authors:** Enza Gucciardi, Sherry Espin, Antonia Morganti, Linda Dorado

**Affiliations:** School of Nutrition, Ryerson University, 350 Victoria Street, Kerr Hall South, room 349-I, Toronto, ON M5B 2K3 Canada

**Keywords:** Canada, Collaboration, Diabetes education, Diabetes management, Integrated care, interprofessional, Collaboration, New work, Primary care, Specialist care

## Abstract

**Background:**

Specialised diabetes teams, specifically certified nurse and dietitian diabetes educator teams, are being integrated part-time into primary care to provide better care and support for Canadians living with diabetes.

This practice model is being implemented throughout Canada in an effort to increase patient access to diabetes education, self-management training, and support. Interprofessional collaboration can have positive effects on both health processes and patient health outcomes, but few studies have explored how health professionals are introduced to and transition into this kind of interprofessional work.

**Method:**

Data from 18 interviews with diabetes educators, 16 primary care physicians, 23 educators’ reflective journals, and 10 quarterly debriefing sessions were coded and analysed using a directed content analysis approach, facilitated by NVIVO software.

**Results:**

Four major themes emerged related to challenges faced, strategies adopted, and benefits observed during this transition into interprofessional collaboration between diabetes educators and primary care physicians: (a) negotiating space, place, and role; (b) fostering working relationships; (c) performing collectively; and (d) enhancing knowledge exchange.

**Conclusions:**

Our findings provide insight into how healthcare professionals who have not traditionally worked together in primary care are collaborating to integrate health services essential for diabetes management. Based on the experiences and personal reflections of participants, establishing new ways of working requires negotiating space and place to practice, role clarification, and frequent and effective modes of formal and informal communication to nurture the development of trust and mutual respect, which are vital to success.

## Background

Currently, 2.4 million Canadians (approximately 7 percent of the total population) are living with diabetes, and by 2019 this number is expected to increase to 3.7 million [1]. Approximately 40% of people living with type 2 diabetes develop long-term, potentially fatal complications including microvascular (e.g. retinopathy, neuropathy, and nephropathy) and macrovascular (e.g. peripheral and cardiovascular) conditions [2–7]. However, long-term complications can be delayed or prevented with appropriate self-management and treatment [8–12]. Diabetes self-management focuses on self-care behaviours to reduce the risk of complications, including healthy eating, physical activity, blood glucose monitoring, medication management, and foot care [13]. Given the complex nature of the disease, a variety of health professionals (e.g. dietitians, nurses, podiatrists, endocrinologist, exercise professionals, and ophthalmologists) can help manage diabetes under the coordination of the primary care physician; an interprofessional team approach is known to be essential for diabetes management [14]. However, the majority of Canadians living with diabetes are still cared for solely by primary care physicians [15].

Within their scope of practice, diabetes educators often spend more time than general practitioners, and have more specialised skills in consolidating the patient’s knowledge and skills regarding eating plan, physical activity, self-monitoring, medication usage, initiation and support with insulin therapy, and training in foot care. Diabetes self-management education primarily delivered by diabetes educators is reportedly effective in improving self-care behaviours [8–12, 16], glycaemic control, lipid profiles, and blood pressure, thereby reducing both the risk and progression of diabetes-related complications [3–7, 17] . However, diabetes education programmes are underutilised by patients [18, 19], probably as a result of various systemic and functional barriers [20, 21].

Patient care can be compromised when health practitioners lack access to the full range of skills or technology necessary to fully achieve their therapeutic goals [22]. In this context, they must refer to other practitioners to achieve their therapeutic or treatment goal; this is referred to as a therapeutic partition [23]. The consequence of therapeutic partitions is that patients must engage in multiple clinical transactions to achieve a single therapeutic goal. Therapeutic partitions can involve more expense [24], more time, and can create vulnerabilities in care delivery [25] compared with an intervention provided by a co-located team [23]. From a patient perspective, truly accessible care requires the provision of appropriate health care in the right place at the right time [26]. Organisational and service delivery restructuring is needed, but few studies have explicitly examined the growing approaches designed to streamline and integrate health professional services in primary care.

Integrating mobile diabetes education teams into primary care is based on a model involving the use of an interprofessional collaborative approach. Each team includes a certified diabetes educator nurse and a dietitian who provide diabetes self-management training and support to patients with type 2 diabetes, and to their primary care providers, in primary care settings.

However, inter-disciplinary care involves increasing interdependence between different types of service providers, and few studies have explored how this is effectively translated into practice in diabetes primary care. The literature on similar integrative models used in primary care has primarily examined patient metabolic outcomes, with little focus on the transition into a new way of professional working, collaboration between health professionals (i.e. primary care providers and diabetes educators).

Our study explored how health professionals experienced interprofessional collaboration (“a type of professional work which involves different health and social care professions who regularly come together to solve problems or provide services” p.45) [27]during the integration of diabetes teams at various primary care sites. We used their experience as a lens through which to understand the structural and practical barriers and enablers associated with the introduction of this new way of working. The findings can help guide future implementations of such a model in primary care, by identifying strategies to improve the transition among healthcare providers and help them provide the best care for patients with diabetes.

## Methods

### Integration of diabetes teams in primary care

Each diabetes team was comprised of a nurse and a dietitian-certified diabetes educator. Teams primarily provided patients with self-management education, coaching, timely treatment adjustment (access to remote glycaemic regimen optimisation and monitoring via telephone and email), and system navigation support. They also provided medication optimisation recommendations and decision support for diabetes management to primary care physicians in primary care settings. Educators were on site either weekly or monthly, depending on patient case load.

Patients were referred to the diabetes teams by their primary care physicians. The intervention was primarily targeted to reach patients with type 2 diabetes who were newly diagnosed, and were experiencing poor glycaemic control, diabetes complications, or needed insulin initiation. Because patient referrals varied across sites based on physicians’ discretion and the site partnership agreement with the diabetes education programme, some diabetes teams also saw patients with insulin glucose intolerance and type 1 diabetes; but the majority of patients had type 2 diabetes. Patients who typically require intense and specialised treatment, such as some with type 1 diabetes, gestational diabetes, or those on a multiple daily insulin regime, were also referred to a diabetes education programme.

The diabetes teams saw patients (for half an hour each with an RN and an RD, or together depending on space availability) to assess each patient’s level of diabetes self-care, diabetes knowledge, and lifestyle habits. The diabetes teams provided individualised patient education and developed treatment priorities and action care plans in consultation with the patient; these plans were shared with the primary care provider, who reinforced them on subsequent visits. If all care providers were concurrently on site, case conferences were conducted when major changes to the patients’ treatment plan (e.g. insulin initiation, prescription for supplies, dose titration) were considered; thus, the primary care providers and educators collaboratively managed patient care. All patients were also encouraged to attend local diabetes education programmes for additional support services (e.g. education classes, workshops, cooking demos, grocery store tours). Half-hour follow-up visits with the diabetes teams were scheduled over a one-year period for all patients, during which action plans, patient goals, and needs were reviewed, discussed, and possibly revised. Additional follow-up visits took place after the first year based on patient needs and the educator’s clinical judgment, such as when a patient’s HbA1c was outside the target range, a patient required insulin start or insulin adjustments, or a patient requested more visits.

### Study locations

Mobile diabetes education teams were sent to 11 primary care sites in a region of Ontario, Canada, between November 2009 and August 2014. Of the 11 primary care sites, eight were family health teams (physicians working in interdisciplinary teams but not including diabetes specialists), two were family health groups (three or more physicians practising together – not necessarily in the same office space but in close proximity), and one was a solo physician practice. Sites were selected based on the established relationships between the diabetes education programmes and the primary care sites, or providers were willing to integrate diabetes teams onsite.

### Data collection and participants

Three types of data were collected from the diabetes educators regarding their experiences implementing the intervention: (a) 18 in-depth, semi-structured, face-to-face interviews with 8 nurses and 10 dietitians (including a clinical team lead); (b) 10 quarterly group debriefing sessions with diabetes teams; and (c) 23 voluntary monthly reflective journal entries across all sites. In-depth interviews were also conducted with 16 primary care providers (half of those participating) by phone or face-to-face. All interviews were conducted at least one year after the intervention began at each primary care site. Demographic data including care provider age and number of years practicing were collected (refer to Table 1). Interview times ranged from 45 min to 1.5 h. Purposeful sampling was used to select diabetes educators and physicians for interviews from all the participating sites. Patients were purposefully sampled to represent a range (1–10) of visits. Patients who had at least one appointment with a diabetes team were invited by their educators to be interviewed. For each participant group, interviews were performed until saturation was achieved (i.e. no new themes were being generated) [28].

**Table 1:** Demographics for patient, primary care provider and educator Interviewees

Variable	Primary Care Provider (*N* = 16)	Certified Diabetes Educator (*N* = 18)
Age groups		
30–39	7 (43.8 %)	4 (22.2 %)
40–49	3 (18.8 %)	5 (27.8 %)
50–59	2 (12.5 %)	8 (44.4 %)
60+	4 (25.0 %)	1 (5.6 %)
Sex		
Female	7 (43.8 %)	18 (100 %)
Male	9 (56.3 %)	0 (0 %)
Highest level of education	N/A	N/A
Less than high school		
Highschool/GED		
Vocational/technical school		
Some college		
Graduated college		
Graduated university		
Number of years living with diabetes	N/A	N/A
Number of years practicing	18.1 ± 12.6	12.75 ± 6.2

Interview guides were developed for each group of participants (refer to Table 2). Interview questions were developed by the research team to elicit responses that describe how care providers were working together. The questions were piloted with two participants from each group to assess clarity, comprehensiveness, and ease of completion. Diabetes teams were also asked to attend quarterly debriefing sessions to discuss their experiences and any implementation issues that arose, and to maintain reflective journals. A monthly email reminded educators to submit a reflective journal entry that they wanted to share. Journal data were transmitted via a confidential online form (Opinio), via a Word document, or during an audio-recorded meeting with the research coordinator.

**Table 2:** Interview guide

Core questions for Diabetes Educators and Primary Care Providers
How does the MDET model facilitate how you care for and support your patients?
How did you feel about the team work/process?
Were there any specific changes to the way you practiced/delivered diabetes care to patients?
Describe your ability to build a working relationship with the PCPs. Describe your collaboration with the dietitian. Describe your collaboration with the nurse. Any other health professional?
Describe your experiences using the patient communication tool. Describe its utility. How do you communicate with the educators regarding patient information (EMR, patient care conferences, any other communication tools)? Are there any barriers to communication? Or any methods or tools that facilitate communication?
Describe any need for resources and/or training that would have improved the implementation of this intervention.
Would you describe the intervention as a success or failure (and why)? Describe some of the factors that made the implementation of this intervention successful.
Describe your thoughts on the patients’ experiences of having diabetes education delivered in the physicians’ offices. What were the advantages or disadvantages?
How can we deal with the challenges/barriers you mentioned to improve upon the MDET intervention?
Are there any other issues you would like to discuss about the intervention?
Extra Diabetes Educator Questions
How do you feel you have contributed to the PCP’s knowledge & management of diabetes care?
Describe the PCP’s accessibility when you needed to speak with him/her about a patient.
Extra Primary Care Provider Questions
How was the MDET introduced to you?
Who do you refer to the MDETs? Why do you only refer these patients and not others?
When you don’t refer to the MDET, do you tell the patients about the program or other resources available to them?
Of the patients that you refer, are there any who refuse to go or are scheduled and don’t show up? If so, do you know why?
Describe your experience of having a MDET onsite. What are the advantages or disadvantages?
Describe your experience with insulin initiation for your patients since having the MDET onsite.
Describe your experiences in responding to RN/RD recommendations (e.g., for medication changes, timely manner, quicker response). Do you normally see your patient the same day that the MDET team sees your patient?
Would you recommend participating in this intervention to your peers? Why or why not?

The study protocol, consent forms, and interview guides were approved by the institutional research ethics review boards at Ryerson University (REB 2010-282-2) and the participating hospitals/facilities. After the study was described to participants, written informed consent was obtained. All interviews and debriefing sessions were audio-taped and transcribed verbatim.

### Data analysis

Data were analysed using a directed content analysis [29, 30]. This analytical approach involved three researchers reading the reflective journals, transcripts of the debriefing sessions, and interviews line-by-line to identify codes. The team then met and developed an initial list of codes by consensus. These codes were then grouped under categories (sub-themes), which were then collapsed into three broader themes. This was an iterative process whereby the team members would review transcripts and the emerging coding schema separately, and then meet to refine the coding schema until consensus on themes and sub-themes was reached. To ensure methodological rigor and trustworthiness of the data analysis, the research team developed an audit trail including the triangulation of responses from in-person interviews, reflective journals, and debriefing sessions to the open-ended questions and the summative content analysis. NVivo software (version 11) was used to facilitate coding across all datasets. The study participants are identified in the results section according to their profession: diabetes educator (DE) and primary care physician (PCP).

## Results

The results are organised below based on four broad themes that emerged from the data analysis. These four themes describe the attributes and professional contributions that appeared to facilitate a more or less functional approach to the new form of working collaboratively in primary care by primary care providers and diabetes educators: (a) negotiating space, place, and role; (b) fostering relationships; (c) performing collectively; and (d) enhancing knowledge exchange. Specific quotes are included below to provide meaning and context to participants’ experiences.

## Negotiating place, space, and role

### Navigating the environment

The first theme involved the experiences of educators in adapting to a new environment. Diabetes teams attended the primary care sites for half a day to a whole day, varying from once a month to weekly. During the early stage many educators felt like ‘outsiders’ and tentatively balanced the necessity to express their needs and attempt to build rapport to effectively do their job while simultaneously avoid adding burdens or hindering the daily routine of the site providers and staff. Therefore, they often avoided initially pressing issues (e.g. appropriate scheduling, space resources, and access to electronic medical records) until they had demonstrated the benefits of the intervention or felt more comfortable initiating these types of conversations. Once educators felt their needs were accommodated and work supported, they felt more at ease working at these sites. The following quotes illustrate the sentiments of educators at the onset of the intervention:

*DE2: I felt – we felt – really kind of like…*

*DE7: You’re an outsider.*

*DE2: Yes, we’re literally like walking on tippy-toes.*

*DE11: Initially we were developing the programme, we were in to get more doctors. So we were just trying to compromise. But now I think since we have grown so much, so I think we are good enough and I think now we can set our priorities, you know, what we are actually looking for.*

### Adjusting role/attitude for success

In addition to navigating the new environment, educators had to ‘adapt’ to the role of working in primary care. The characteristics perceived as necessary to be a valuable team player, by both educators and primary care providers, were being ‘flexible,’ ‘easy-going,’ and ‘confident.’ Primary care providers suggested that educators could be more forward in engaging with them, and referred to the importance of taking the initiative to be successful in an unfamiliar worksite:

*DE2: [You] need to make sure that you’re a good working part of the team. That you’re not going to the physician with frivolous things and that your recommendations are clear and they are concise…that they make sense…. Like you’re there to add value – I mean, they are giving up an office space for you, which is huge, right? And then I guess…you go with the flow. That you not sort of be upset about the fact that you might be asked to move offices three times in one day, or whatever.*

*DE7: I think you have to be an educator who’s very flexible, easygoing, take things that are said to you…. You can’t be defensive that you’re not doing a good job. You need to work with them and find out, okay, how can I make this better for your patient and for you…. ‘Cause really, we’re there for the patient to get knowledge about diabetes and for us being there, the accessibility is an important part of it, right? You just have to go over and try to be very diplomatic when you’re stating things…. So I think at the beginning there’s a personality thing too. So, not everybody’s going to be perfectly matched. That’s common everywhere. So you just have to learn to work together and network together.*

*PCP1-2 You’ve gotta have staff who have the right personality as well, that are comfortable to go into that environment…. She [DE7] was never really kind of afraid to knock on doors and ask questions, and so on. But you need to establish that relationship.*

## Fostering working relationships

The second theme that emerged from our analysis involved the significance of building working relationships among the clinicians involved in the intervention at each site over time. Aspects of this theme included understanding scope of practice, roles, and responsibilities; and degree of familiarity and informal interactions.

### Understanding scope of practice and specialisation

One critical component of facilitating a collaborative working relationship among health professionals is having a common understanding of each professional’s scope of practice and responsibilities in caring for the patient. Educators indicated that meeting prior to and/or at the start of the intervention and establishing service agreements to clearly define the diabetes team services, roles, and responsibilities helped improve working relationships with primary care providers and the support team at each site, such as office managers and administrators. Cases of misuse and underuse of educators were observed throughout the intervention, possibly due to poor orientation processes and a lack of clarity surrounding educator scope of practice. Examples included overbooking patients, leaving insufficient time to spend with each patient, booking patients without diabetes, and a lack of referrals to their services overall:

*DE5: It really does ultimately come down to the relationship that you have and the communication that you have with the other people that are involved in a patient’s care to make that patient’s care better…. The service agreement’s a huge help to that, ‘cause I think if everyone knows what to expect…‘cause we’ve had situations where the service agreement wasn’t necessarily followed and then it makes it much more challenging for the staff. So they end up with 10 or 15 patients a day instead of 5 or 7…. We’ve formalised some of those things and we go in ahead of time and make sure that…it works a lot better.*

DE3: *… I’ve been [to] about four or five offices now – and I find that…the experiences are very inconsistent from facility to facility.… Probably the biggest challenge, I find, is in reinforcing boundaries, because some of the offices, once they have us [the diabetes specialists/team], it’s that, ‘Oh, well, you’re here, you have the right letters next to your name. Can we squeeze in this person that doesn’t have diabetes? They’re anorexic,’ or ‘They have dyslipidemia’…. It’s happened in other offices as well. Or they are overweight, and they’re 14 years old…. There’s also been, in several of the offices, a certain pressure to work faster and to see a higher volume*.

The degree of collaboration varied across sites. Some educators perceived less interaction or ‘co-management’ between primary care providers and dietitian educators compared with primary care providers and nurse educators, primarily due to overlapping or non-overlapping scopes of practice, or shared role boundaries. This finding is supported by the following quote from a primary care provider:

*PCP 1-2: I think that there’s a lot more co-management between the physicians and the diabetes nurse educator in terms of, you know, what’s the plan and changing medications and prescriptions, and all those kinds of things that we need to work together more closely. Whereas, the dietitian is able to kind of manage the whole dietary piece on her own. She doesn’t need to necessarily collaborate and consult with me in the same way. She needs to report the main themes of their conversation, and if there’s any goals that have been set and so on, but there doesn’t need to be that sort of deeper collaboration that’s required around medication management.*

### Degree of familiarity

Professional relationships between diabetes educators and primary care providers that had been established prior to the initiation of the intervention evolved quickly and easily into successful working relationships, facilitating confidence, trust, and value in educator competency among primary care providers. Educators and primary care providers considered these components to be critical:

*Clinical Team Lead: DE7 had a working relationship with the two doctors– [PCP 1-1] and [PCP 1-2]. She had worked with them when she was an OR nurse here. So she actually had, like, a 20-years ago working relationship with those physicians and knew them. So our interaction with them right from the start was [based on] a trusted relationship with [DE7] already. So there really were no barriers.*

*PCP 1-2: I think there’s a factor that helped make it successful, which is that I have familiarity with the diabetes nurse educator…and I think that does make it easier when you have actually had some interaction with the person before because…your ability to function as a team is off to a head start compared to [with] a stranger– you’re not sure about where they’re coming from, they’re not sure where you’re coming from, you don’t know each other’s skill.*

In the absence of a pre-existing relationship, a crucial component to enhancing working relationships appeared to be developing trust and rapport among team members over time, by meeting regularly or even simply being on-site at the same time as primary care providers. Two educators explained how this can occur:

*DE17: I think maybe…some of it takes time and trust. Right. That rapport for the physicians to really…they are officially letting you into the circle of care before they trust your recommendations and feel comfortable with it. It’s just [takes] time….*

*DE4: I think meeting with the doctors, all of them, is really important….‘Cause I think if they trust the people [diabetes educators] that go there, that the patient really benefits, ‘cause the patients will be referred and the patients will see that, yes, the doctor respects our expertise [diabetes team] and supports it.*

Proximity was also cited as an important factor for the referral of patients to the diabetes team. Educators explained that they tended to get the most referrals from primary care providers who were at the site on the same days as themselves. In a reflective journal entry, one educator wrote:

*DE9: I am finding that the doctors who are referring are more often the doctors who are working the afternoon we are at the office. I feel it’s because they see us on a regular basis and we get a chance to speak with them more often than the other doctors in the practice. We have had meetings in the past to introduce ourselves and again discuss what we do with all the doctors but still the ones we don’t see often are less likely to refer.*

### Informal interactions

Enriching relationships appeared to be related to proximity and communication via informal channels, e.g. personal interactions when having lunch together, talking in hallways, invitations to primary care site events and gatherings, or personal inquiries from primary care providers on behalf of their family members living with diabetes. The following quote illustrates how these personal exchanges are valued:

*DE4: I think that having them [PCPs] right there and using their offices, and having lunches together and, you know, seeing each other in the hallway – all of that closeness, it helps. It’s a big place but then it’s not. It’s a small office with everybody there, so that also helps build the rapport amongst each other.*

Although informal interactions were identified as important in building a more collaborative practice, they did not occur very often at primary care sites. Reasons cited for this included the fact that educators and primary care providers were often not on-site at the same time, or that educators were at the primary care site infrequently, diminishing the opportunities for interpersonal contact. At some sites, even those where educators were present weekly, they still did not feel a part of the primary care team and tended to feel excluded from more social functions and interactions. For example:

*DE22: When you’re travelling from site to site, it’s really lonely, okay. You don’t belong anywhere. And that makes it very lonely, so you don’t…your [diabetes educator] partner....is your only sort of, you know, this is the person that you’re going to spend the bulk of the day with, that you’re going to talk about…things, you know, any sort of issues that are bothering you…. This is your, this is your team, because you can’t go to a site and just talk to somebody there about something that’s bothering you ‘cause they don’t really care. They don’t understand.*

*DE22: At Christmas time, sometimes we get included in the department party, sometimes we don’t because they do not think of us as part of their team.*

## Performing collectively

This third theme involved how educators, primary care providers, and primary care staff worked together to deliver comprehensive and integrative patient care. Sub-themes included face-to-face interaction, non-face-to-face communication, co-management of care, and stability of team members.

### Face-to-face interaction

Implementation of the pilot study required new ways of working together, including new ways to share information. Primary care providers and educators described how they worked collaboratively to care for patients and relay treatment recommendations and management decisions. Face-to-face interaction among all team members appeared to facilitate timely responses, particularly with primary care provider follow-through on patient recommendations and prescription orders from educators. The following quotes illustrate this point:

*DE5: If we do make any suggestion about changes or recommendations, we can always get [the patient’s] physician right there and then. And then they will be able to go away either with a new prescription or new things to try, because their doctor’s right there for us to consult. So I think…it all works in their [patient] favour*.

*PCP 1-2. Most of the time, I’m here when they’re here. So [TE7] will just say, ‘I think we should do this or that,’ and I’ll almost always agree, and it’ll be done. So usually the response is within minutes. I suppose rarely something happens when they’re not on site and then usually there’ll be a communication one to another, we’ll communicate with each other and then give the okay*.

*PT8: And if there’s any onset of something they (diabetes educators) can question [it] or that they think it isn’t right, they can go straight to the doctor and say, ‘PT8 is here, but you know this is happening and that’s happening and it’s not right.’ You know…whereas before there would be a note or an email sent to a doctor and then the patient leaves.*

*PCP 15-2: In the past, when they were on site, we [would] do a lot of hallway consults or really brief meetings. But I think that there’s a real advantage to doing that in-person with certain patients.*

Primary care providers and educators referred to these face-to-face interactions as ‘corridor consultations’ or ‘hallway consults,’ and referred to their benefits for delivering better patient-centred care. Primary care providers found this to be especially true for patients with co-morbidities, those from marginalised population groups, or those coping with social issues. Additionally, face-to-face interactions were considered beneficial for the transfer of vital patient information that may not have been recorded in the patient’s chart. Diabetes care was coordinated efficiently for patients even in the occasion of an absent physician, by relying on the team of physicians working together at the site:

*PCP 7-5: To have them here, I can speak to DE22 and DE23 about [a particular] patient face to face, and she would say, ‘Well, I think he’s on a prescription for some lantus, or I think he needs more glucometer sticks, or you know we’re going to do this and this and see me again in a few weeks and that’d be great.’ So there’d be a sort of an interaction, an exchange of information which was very helpful.*

*PCP 7-5: Every time I walk by DE23’s office, she'll come over and tell me about, ‘So-and-so is not doing well.’ Or maybe there’s some other problem going on that I wasn’t aware of, for example, ‘He’s got this rash, or he’s got, you know…chest pain or something like that,’ that I need to be aware of. So yeah, I mean the communication is open and available and I think it's good. I think it’s another benefit of them being on site here.*

### Non-face-to-face communication

Some primary care sites used indirect methods of communication to relay information about their patients, principally because the diabetes team was only on-site when office space was available due to the absence of a primary care provider. A few diabetes educators said that EMR notes were one way that they could work together with the primary care provider, as certain primary care providers would follow-up with patients on specific care recommendations such as physical activity, carbohydrate counting, or blood glucose monitoring. However, at certain sites educators did not have access to electronic medical notes, so they resorted to ‘workarounds’ such as email messages, handwritten notes, and/or communicating through support staff. Educators said these methods of communication were still somewhat effective in facilitating the exchange of patient information and patient care. For example:

*DE5: Because it wasn’t like we don’t communicate.... The recommendation that we made is being followed through. Or even if there’s no blood work, then the next time there’s some blood work. So it is communicated, I think, in some way – unless the physician does not like us doing it, we will hear about it. And they are there if we need any, you know, major changes. If we need blood work or if we need medication changes, they’re there. But it’s not like they, you know, actively participate. If everything goes smoothly, they don’t participate. They don’t come in and get briefed on everything that we do.*

### Co-managing care

Primary care providers reported that they could work with educators to reinforce a consistent message for their patients, which was important for motivating lifestyle management change or commencing insulin initiation. They also appreciated the support and reassurance from educators with regard to their treatment plans for patients:

*PCP 7-5: I could reinforce whatever DE22 said, like patients don't want to go on insulin with diabetes, like the big issue with type two is that they’ll often need insulin, and be, ‘Oh, I don’t want the needle, I don’t want the needle.’ But then, you know, I say, ‘You know, the needle is actually going to make you feel a lot better and, you’re sugars are going to improve.’ DE22 gives the same message and then I give the same message again. So we reinforce each other on the message and it’s consistent and strong and the patients, I think patients benefit from that consistency.*

*PCP 6-3: [Duplicated messages] reinforce what I’m telling them, in terms of diet advice. So it helps the patient to hear it coming from two different sources. So compliance is better. And then generally it’s always good to have someone else look at the chart, see—it’s like another pair of eyes looking and saying, ‘Yeah, so and so should be on this. This is good.’ It’s just confirmation and reassurance that either you’re doing well or maybe a friendly suggestion to change.*

One primary care provider explained how educator support was important for insulin initiation:

*PCP 15-2: Because the reality is, we can tell them, ‘You have diabetes, you need to be on insulin because of your numbers or something.’ But, until they feel supported in terms of starting a new lifetime treatment, like insulin injections, they’re never going to start, and I think that that’s the tipping point of where the diabetes team really makes a big difference. Because it’s that extra level of support that we [physicians] might not be able to provide, as a primary care physician, just because of time constraints and stuff. And, also, our own primary care nurses upstairs might not be able to provide this care because of the expertise that DE22 and DE23 have.*

### Stability of team members

Another factor that facilitated collaboration was having the same educators returning to the same primary care sites. Educators preferred working with the same educator partner when they saw the same patients; they relied on each other for support. In general, educators reported that both patients and primary care providers preferred to have the same diabetes team because it promoted familiarity between the primary care provider and their patients, and contributed to consistency in care. Patients preferred to develop a relationship with educators and appreciated being cared for by a familiar team of healthcare providers. The following excerpts illustrate this point:

*DE4: Patients, they like seeing the same [educator]. You know, they go to that office and they always see the same doctor so they expect the same from us–that they will see the same person rather than seeing all kinds of new people…. They want some consistency. So we’re trying to provide that now–if we are on vacation, then one of us will go and they will still have that familiar face, somebody that they know already and they know they can trust.*

*DE4: Well, it’s actually better because…there [at the PCP site], you have this one designated RN that you always work with. And she is seeing everybody that I’m seeing. So actually, I think that really improves the teamwork…over there [PCP site], it’s her and me…. And she’s familiar with all the patients that I’m seeing.*

Additionally, Primary care providers felt their patients were in ‘good hands’ with educators and preferred being able to make a referral to educators with whom they had developed a working relationship, as opposed to a referral to the diabetes education programme, which often had a long wait list and unfamiliar educators.

## Enhancing knowledge exchange

The fourth theme that emerged from the data involved knowledge exchange between diabetes educators and primary care providers and their staff. The intervention not only created an opportunity for clinicians to discuss their patients in real-time, but to also share information, such as a patient’s story, that may not appear on a medical chart. Furthermore, due to their experience in managing diabetes, educators could sometimes access information from patients that the primary care provider was unaware of. Educators also updated primary care providers about the latest treatment modalities or practice guidelines for diabetes, and the diabetes support resources available in the community. For example:

*DE17: The (PCPs) tell the story, they (PCPs) come to us before we see the (patients) to say, ‘I think it’s good that you understand the story.’ And I think those are doctors who have a really good relationship with their patients. I’ve heard a patient say, ‘That’s why I’d rather see you here because I know now that you’re working as a team. I don’t feel like I have to go to a new place and tell my whole story all over again. I feel confident about that.’*

*PCP 8-1: Sometimes, DE22, or DE23, will tease out something about what’s going on with the patient’s self-administration of meds or diet that I’m not getting, and they’ll say, ‘Their needs and A1Cs are this,’ that the other thing may be because of the fact that the person is doing one thing or another that I would not have picked up on. Or they’ll say, ‘You know, we found in this situation…’ –‘cause they’re dealing with lots and lots of diabetics–‘that this manoeuvre tends to work better than that manoeuvre, or this tends to happen because that tends to happen.’ So, in essence, they’re getting, we’re getting the benefit of them seeing larger volumes of purely diabetics, so they get very skilled at knowing the ins and outs.*

Educators used various methods to transfer specialised diabetes knowledge to primary care providers and their staff. For example, one diabetes team developed and trained a nurse resource person at their site to sustain the intervention in their absence. One diabetes educator and primary care provider relationship evolved during the intervention, such that the primary care provider began calling the educator at the diabetes education programme to discuss other diabetes patients, who the educators had not yet seen. Educators also felt that electronic patient notes were a good way to transfer specialised diabetes knowledge to primary care providers, and made a point to write detailed notes regarding the content of the educator sessions and patient treatment recommendations:

*DE9: Sometimes, I will look at it as an educational thing, even for summaries here. Because sometimes if I make that recommendation of, you know, especially if they are not having enough carbs, I will be specific and say, ‘Minimum, make sure they’re having a minimum of X amount of carbs at meals or for the day,’ so that the doctor recognises that, you know, there may be a concern.*

Educators identified diabetes knowledge gaps among primary care providers, including diet regimens, A1C guidelines, insulin initiation, and new medications. Our findings revealed considerable trepidation among primary care providers regarding insulin initiation. They felt more confident about insulin initiation when sharing knowledge and collaborating with educators:

*DE16: The (PCPs) are fearful, they’re anxious, right? And they don’t know how to proceed–inertia, there’s a clinical inertia. So we’re helping to reduce that clinical inertia with, you know, ‘Let’s get this guy on insulin, or let’s increase the dose, or change the dose or….’ So I think with that piece, the clinical inertia, we’ve had significant impact. And I’m talking all sites.*

*PCP 7-5: It’s been easier for me to initiate insulin with DE22’s expertise and…she’s taught me, you know, some of those, the fine points of doing that and, yeah, it’s an ongoing input. I mean I knew how to start insulin before…[but] she’s definitely improved that skill in me.*

Primary care providers repeatedly referred to the value of the educators’ expertise and its benefits when making clinical decisions. They described their shared decision-making approach as a ‘meeting of the minds’ rather than an ‘off-loading’ of patients to the diabetes teams; one said it was “really a back and forth” process:

*PCP 15-2: The reality is–for someone who doesn't do a ton of insulin starts–that was really useful for me. And my sense is, for a lot of family physicians who don't do a ton of insulin starts, where they can [work] with someone like DE22 who’s quite experienced in that, it works so much better. Like we can have theoretical knowledge…but I think the practical part of it is the missing link, and that's where I think ME1 makes a difference.*

## Discussion

In Canada, it has become common to overcome inefficiencies in delivering diabetes care in primary healthcare settings by better coordinating care and creating integrated service models with diabetes specialised teams. However, the complex negotiation of space, roles, and relationships can be challenging when hierarchies continue to persist within the healthcare system. Our findings help clarify how health professionals establish themselves within a new work environment, new work role, or given new work partners. Specialised diabetes teams entering existing primary care settings are inevitably faced with challenges related to the need to fit into an already functioning and traditionally hierarchal system. Some of these settings require diabetes educators to adapt as newcomers to a different organisational structure that may not easily lend itself to flexibility and interprofessional collaboration [31]. This is compounded when there is a lack of preparation: some sites were integrated without formal orientation procedures, making the new role appear as if ‘dropped out of thin air’ [32]. Diabetes educators characterised themselves as ‘outsiders’ in the primary care setting, and a disconnect was observed at many sites between valuing the role of educators and accommodating their needs to ensure the functioning of their practice. Educators had to make an effort to assert their role and carve out a place for themselves due to the lack of active integration; Baker et al. described this as ‘elbowing behaviour’ [33]. A demonstrable level of achievement was often needed to garner the recognition and support to create an effective work environment for the diabetes teams. Trust was often lacking among new team members because their professional competency and ability had yet to be demonstrated [27].

Previous research has suggested that the introduction of new working relationships may not always be successful given the lack of demonstrable achievements, but also from poor role definitions and poor relationships [32, 34]. According to Whiteford et al. [28], the most important factors for promoting effective service integration are ensuring understanding professional roles, mutual respect, and efficient communication among all involved in the care and support of patients [35]. Various interprofessional competency frameworks designate role clarification as a key feature for developing strong interprofessional relationships [36–38]. Role clarification consists of practitioners demonstrating recognition and respect of fellow practitioners’ scope of practice [39]. Conflict and lack of trust can occur among team members when health professionals do not understand each other’s roles and their application to patient care or when skills overlap [40]. Diabetes educators often attribute misuse (i.e. overbooking patients, insufficient appointment length, and referral of patients without diabetes patients) or underuse of their services to a lack of role recognition from collaborating primary care providers and their administrative staff. To avoid role conflict, a formal orientation at the beginning of the intervention and regular team meetings can help health professionals negotiate a mutual understanding of their roles, and functions as a base from which to build a working relationship and develop common goals [40] and a common service model [41]. Micro-interactions during these meetings can help build personal and collective practice; Freeman termed this ‘learning by meeting’ [42]. Our findings also confirm that stability and physical proximity of team members not only provide continuity of patient care, but may also offer opportunities to develop professional working relationships because of the accessibility of team members on site [43].

Our participants’ responses suggest that familiarity, proximity, and informal interactions facilitate the sharing of information about one another, which is known to enable collaborative work practice [43]. Furthermore, the type and regularity of communication plays a crucial role in how new team members work together [40, 44, 45]. Studies have shown that informal contact is necessary for promoting intergroup relations and understanding of each profession’s approaches and professional priorities [46]. Trust develops as familiarity grows among the members of the healthcare team, potentially blurring professional boundaries and hierarchies and encouraging collaboration. According to the intergroup contact theory, contact between members of different groups can enable discovery of mutual similarities, which can dismantle perceived barriers to relationship building and generate positive change to potential stereotypical attitudes [47]. Shepherd and Meehan referred to interpersonal communication as the ‘glue’ of interagency collaboration [48]. However, some diabetes educators reported that a lack of informal relationships made them feel disconnected from the primary care site team.

Effective work communication among new team members is also integral to the success of a collaborative model of practice and can take many forms, including verbal and non-verbal interactions [27, 46]. Our care providers reported that face-to-face communication allowed for more timely follow-through of recommendations such as ordering tests, prescriptions, and medication changes, quite often while patients were still on site. Although most interviewees acknowledged that real-time communication was preferable to address patient care, this was not always feasible in many of the primary care sites. In many settings, professional relationships and collaborations were realised through the use of electronic medical records, emails, and administrative staff; and primary care providers still followed through with recommendations made by educators, but these indirect modes of communication are considered less effective for team functioning [49]. Regularly scheduled meetings, case conferences, and ‘team huddles’ may better optimise the efficiency of teams by ensuring enough time is allocated to discuss patients and other operational challenges [49, 50].

The integration of specialised diabetes teams in primary care also presents opportunities for primary care providers to enhance their knowledge and practice in diabetes. In fact, it enhances the capacity for all health professionals to learn with and from each other. Educators serve as experts to assist in patients’ self-management, help primary care providers fill potential gaps in practice alleviating clinical inertia, and train on-site staff such as clinical nurse practitioners. Clinical interactions between educators and PCPs revealed the creation of new knowledge by communicating patients’ stories, demonstrating greater communal understanding and a holistic picture of the patient, thereby enabling the provision of targeted patient care. Furthermore, real-time interaction among team members facilitated the creation of solutions through group effort and reflected interdisciplinary expertise. Finally, having on-site educators facilitated partnership with the diabetes education programme and increased primary care providers’ referrals and patient access to the programme’s resources and services external to the primary care site.

### Limitations and strengths

Limitations of this study include a lack of data from other primary care staff, such as administrative assistants or on-site nursing staff at some sites, who may have also played integral roles in the functioning of new team members. Also, the study was conducted in only one urban region in Ontario, Canada, and therefore may not have been representative of issues in rural or remote regions of Canada. However, the study was conducted across sites that differed in organisational structure. Its strengths included the use of semi-structured interview guides, which ensured consistency and reliability in data collection without limiting the conversational flow or discovery of new themes. Data saturation was reached for all participant groups, indicating that the number of interviews per participant group was sufficient to fully explore each relevant theme. Finally, the study evaluation was relatively large in scope, encompassing multiple key participants’ perspectives across three diabetes education programmes and 11 primary care sites.

## Conclusions

The Canadian health workforce needs to be able to respond to the changing needs and demands of the population. This requires challenging the highly complex and socially constructed ways health services are currently organised. During the integration of diabetes education teams in primary care, specialists and primary care providers were able to perform together in co-caring for patients with diabetes. However, service providers will need to adapt to new changes and challenges that may arise regarding sharing of space, costs, and access to and training in technology. Broader government policy support and direction is needed for these complex implementation tasks, rather than leaving them to local and community organisations. Governmental resources are being provided for collaboration in healthcare, but more financial and educational supports need to be provided in primary care, where interprofessional collaboration is a fairly new process, to enable healthcare professionals to gain the knowledge and skills required for effective collaboration and to ensure new service developments have positive outcomes for both patients and professionals.

## Appendix

**Table 3:** Graphic representation of our audit trail

Original Node	Renamed	New Node	Themes	Final Themes
Scope of practice roles/responsibilities		Understanding Scope of practice, roles and responsibilities	Creating Relationships	Fostering working relationships
Selling MDET to Pts				
PCP billing for Pts				
Inappropriate use of MDET				
PCP interaction with nutrition therapy				
Meeting the PCPs and Staff				
		Informal Relationships		
Information Collaboration	Informal Relationships			
Trust		Degree of familiarity		
Feeling Valued				
Established relationships	Existing Relationships			
Relationship building				
Clinical inertia	MDET as Resource for PCP	MDET Support	Sharing knowledge, resources and support	Enhancing knowledge exchange
Gap in PCP knowledge				
MDET as a Resource for PCP	MDET Support			
Training the NP				
NP Support		NP Support		
DEC Support	DEC Support	DEC Support		
Non F2F Interaction		Non F2F Interaction	Working Together	
Pt. Communication Tool				
Recommendation Follow Through	*Renamed to Written recommendations – assigned quotes to F2F Interaction and Non F2F Communication. Discarded node.*			
PCP unavailability				
F2F Interaction		F2F Interaction		
Immediate Communication and Response				Performing collectively
Formal Collaboration	*Renamed to formal relationships and quotes were assigned to F2F interaction and then discarded.*			
Shared Decision Making	*Some quotes moved into Definition of Collaborative Model node. Need to decide what to do with entire node.*			
Teamwork	Perceptions of teamwork/collaboration?	Perceptions of collaboration		
Definition of Collaborative Model				
Non active participation by PCP or HCP				
Collaboration btw. Educators	Stability of team members	Stability of team members		
Continuity of Care				
Negotiation (1 reference)	Navigating the environment	Navigating the environment	Adapting Space, Place and Role	Negotiating space, place and role
Tip Toe				
Educator Role Expectation	Educator Characteristics for success	Educator Characteristics for success		

## Abbreviations

DE: diabetes educators
PCPs: primary care providers
